# Enantioselective Induction of a Glutathione-*S*-Transferase, a Glutathione Transporter and an ABC Transporter in Maize by Metolachlor and Its (*S*)-Isomer

**DOI:** 10.1371/journal.pone.0048085

**Published:** 2012-10-29

**Authors:** Sen Pang, Zhaojin Ran, Zhiqian Liu, Xiaoyu Song, Liusheng Duan, Xuefeng Li, Chengju Wang

**Affiliations:** College of Sciences, China Agricultural University & Engineering Research Center of Plant Growth Regulators, Ministry of Education, China Agricultural University, Beijing, People’s Republic of China; University of South Florida College of Medicine, United States of America

## Abstract

The metabolism of chiral herbicides in plants remains poorly understood. Glutathione conjugation reactions are one of the principal mechanisms that plants utilize to detoxify xenobiotics. The induction by *rac*- and *S*-metolachlor of the expression of three genes, *ZmGST27*, *ZmGT1* and *ZmMRP1*, encoding respectively a glutathione-*S*-transferase, a glutathione transporter and an ATP-binding cassette (ABC) transporter was studied in maize. The results demonstrate that the inducing effect of *rac*- and *S*-metolachlor on the expression of *ZmGST27* and *ZmGT1* is comparable. However, the inducing effect of *rac*-metolachlor on *ZmMRP1* expression is more pronounced than that of *S*-metolachlor. Furthermore, vanadate, an ABC transporter inhibitor, could greatly reduce the difference in herbicidal activity between *rac*- and *S*-metolachlor. These results suggest that the ABC transporters may preferentially transport conjugates of *rac*-metolachlor, leading to a faster metabolism of the latter. Through comparing the expression of *ZmGST27*, *ZmMRP1* and *ZmGT1* after treatment by *rac*- and *S*-metolachlor, we provide novel insights into the metabolic processes of chiral herbicides in plants.

## Introduction

Chirality is a common phenomenon in life sciences. More than 30% of currently used pesticides are chiral compounds, including synthetic pyrethroids, organophosphate insecticides, imidazolinone and chloracetanilide herbicides [1], [2]. The percentage of chiral pesticides is increasing with the introduction of more complex structures [3]. Enantiomers are defined as molecules that contain chiral structures and mirror images of each other [4]. Enantiomers of chiral pesticides have identical physicochemical properties, but exhibit quite different biochemical activities because biochemical processes usually show high stereo- or enantioselectivity [5]. Over the past two decades, studies on enantimers differing in biological properties have been widely reported [6], including their biodegradation [7], [8], toxicities to non-target organisms [4], [9]–[14], endocrine-disrupting activities [15]–[17], etc. However, the enantioselective physiological effects and toxicities of chiral herbicides in plants have not received as much attention as insecticides in animals [1]. Although the enantioselective phytotoxicity of several chiral herbicides in plants has been evaluated [2], [4], [6], [18], [19], the metabolic processes of chiral herbicides in plants remain poorly understood.

Metabolism of herbicides in plants can generally be divided into three phases. In phase I, the herbicide may be oxidized, reduced or hydrolyzed to introduce or reveal a functional group. In phase II, the herbicide is conjugated to glutathione, glucose or malonate by the respective transferase to form a water-soluble conjugate. In phase III, herbicide conjugates are transported from the cytosol to the vacuole for further degradation. Glutathione conjugation plays a major role in the resistance of plants to herbicides. There have been numerous reports concerning the role of glutathione S-transferases (GSTs) in conjugating xenobiotics and the role of tonoplast ATP-binding cassette (ABC) transporters in transporting glutathione conjugates (GS conjugates) into the vacuole [20]–[22]. In addition to ABC transporters, recent studies showed that glutathione transporters, located in the plasma membrane, are able to mediate the transport of both reduced glutathione (GSH) and GS conjugates [23], [24]. Our earlier work found that the expression of a glutathione transporter gene isolated from maize, named *ZmGT1,* was inducible by herbicides atrazine and metolachlor, and the inducing effect of metolachlor in different maize cultivars was correlated to their tolerance to this herbicide, suggesting an involvement of *ZmGT1* in the detoxification of xenobiotics by plants [25], [26]. Co-induction of *ZmGST27*, *ZmMRP1* and *ZmGT1* in maize by xenobiotics further suggests that, in addition to GSH, GSTs and ABC transporters, glutathione transporters located in the plasma membrane are also an important component in the glutathione conjugation-related plant detoxification system [27].

Metolachlor is a widely used herbicide that inhibits the synthesis of fatty acids in broadleaf weeds [28]. It was first introduced into the market as a racemic product, which contains both *R*- and *S*-enantiomers present in an equal ratio [19]. Racemic metolachlor is currently being replaced by *S*-metolachlor, which contains approximately 90% *S*-isomers and has the same herbicidal effect as the former when used at 65% of its dosage [29]. The sorption and desorption of metolachlor in the soil have been studied, along with its dissipation properties and effects on non-target species [30]–[33]. However, only limited information is available on the enantioselective behavior of *rac*- and *S*-metolachlor in plants [2], [19]. Our previous work tested the effect of metolachlor on the expression of a glutathione-*S*-transferase (*ZmGST27*), an ABC transporter (*ZmMRP1*) and a glutathione transporter (*ZmGT1*) in maize leaves, but the effect of *S*-metolachlor on the expression of these genes was not investigated. Therefore, in the present study we have compared the expression pattern of *ZmGST27*, *ZmMRP1* and *ZmGT1* in maize leaves after treatment by *rac* and *S*-metolachlor. The aim was to provide insights into the metabolism of chiral herbicides in plants.

## Results

### Induction of *ZmGST27*, *ZmMRP1* and *ZmGT1* Expression by *rac*- and *S*-metolachlor

**Figure 1:**
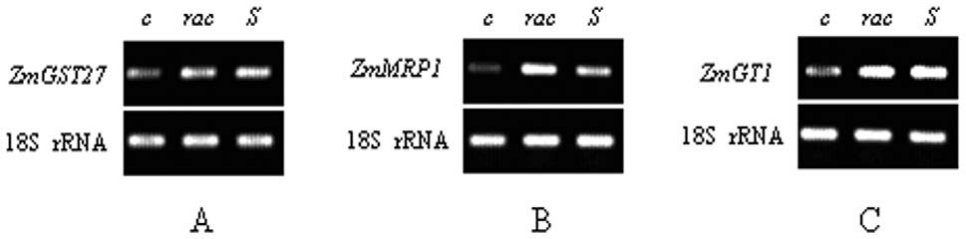
Semi-quantitative RT-PCR analysis of *ZmGST27*, *ZmMRP1* and *ZmGT1* expression in maize leaves at 48 h after treatment by *rac*- and *S*-metolachlor. A: *ZmGST27*; B: *ZmMRP1*; C: *ZmGT1*. *c*: non-treated control; *rac*: *rac*-metolachlor; *S*: *S*-metolachlor. The experiment was repeated twice with similar results.

The semi-quantitative RT-PCR results showed that the expression of *ZmGST27*, *ZmMRP1* and *ZmGT1* was promoted by both *rac*- and *S*-metolachlor after 48 h treatment. While no significant difference was found between *rac*- and *S*-metolachlor in increasing the transcript level of *ZmGST27* and *ZmGT1* (Fig. 1A and 1C), *rac*-metolachlor was found to be a stronger inducer for *ZmMRP1* expression as compared to *S*-metolachlor (Fig. 1B).

**Figure 2:**
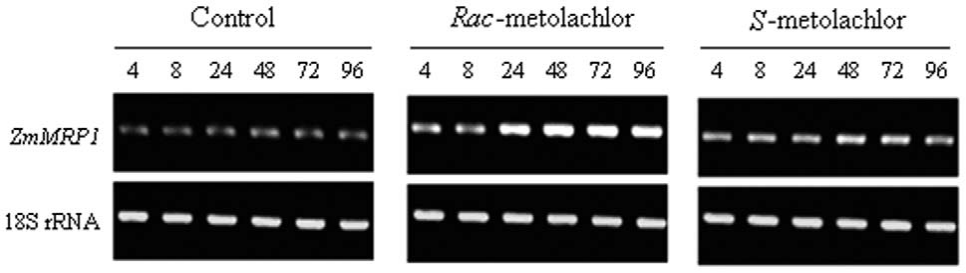
Semi-quantitative RT-PCR analysis of *ZmMRP1* expression in maize leaves after treatment by *rac*- and *S*-metolachlor. The numbers (4, 8, 24, 48, 72 and 96) above each lane indicate the time intervals (h) after treatment. The experiment was repeated twice with similar results.

A time course study was designed to further verify the differential up-regulation of *ZmMRP1* expression by *rac*- and *S*-metolachlor in maize leaves. The expression level of *ZmMRP1* analyzed at 4, 8, 24, 48, 72 and 96 h after treatment by *rac*- and *S*-metolachlor confirmed the initial finding. As shown in Fig. 2, while a consistent but moderate increase in *ZmMRP1* transcript level was afforded by *S*-metolachlor during the entire treatment period (from 4 to 96 h), the induction of *ZmMRP1* expression by *rac*-metolachlor was moderate during the first hours but became dramatic from 24 h of treatment onwards.

### Effects of *rac*- and *S*-metolachlor on the Growth of Maize Shoot

Dose-response experiments in the presence of *rac*- and *S*-metolachlor allowed determination of their respective EC_50_ values on maize growth. The EC_50_ value of *rac*-metolachlor is about 2.1-fold that of *S*-metolachlor, indicating *S*-metolachlor is more efficient in inhibiting the growth of maize shoot than *rac*-metolachlor (Table 1). When used alone, vanadate, an inhibitor of ABC transporters, had no significant effect on the growth of maize shoots (results not shown). However, this same compound significantly enhanced the inhibitory effect of *rac*-metolachlor on maize growth, as judged by the decrease of its EC_50_ from 375 to 184 µM. By contrast, only a small reduction in the EC_50_ of *S*-metolachlor was found when vanadate was applied (Table 1). As a result, the ratio of EC_50_ values of *rac*-metolachlor and *S*-metolachlor decreased from 2.1 to 1.3 in the presence of the ABC transporter inhibitor.

**Table 1:** Effects of various chemicals on the growth of maize shoots (4 d after treatment).

Herbicides	Regression equation	EC_50_ (µM)	*R^2^*
*Rac*-metolachlor	*y* = 0.58ln*x*–3.25	374.51	0.91
*S*-metolachlor	*y* = 0.60ln*x*–3.11	175.41	0.90
*Rac*-metolachlor + vanadate	*y* = 0.45ln*x*–2.34	184.40	0.97
*S*-metolachlor + vanadate	*y* = 0.68ln*x*–3.35	138.58	0.90

* Vanadate was applied at a constant concentration of 100 µM.

## Discussion

Studies on chiral pesticides started to appear in the early 1990s [4]. However, there is still a severe lack of knowledge about the metabolism of chiral herbicides in plants [28]. Metolachlor was introduced into the market in 1976 as a racemic product. It was later found that the two 1′*S*-isomers of metolachlor afforded most of its biological activity. Our earlier work has provided evidence for the involvement of a glutathione-*S*-transferase (*ZmGST27*), a glutathione transporter (*ZmGT1*) and an ABC transporter (*ZmMRP1*) in the detoxification of metolachlor in maize [25], [26]. However, a comparative analysis of the expression patterns of GSTs, ABC transporters and glutathione transporters in the same plant tissue after exposure to metolachlor and its *S*-isomer was lacking.

In this work, we investigated the enantioselective induction of *ZmGST27*, *ZmGT1* and *ZmMRP1* in maize by *rac*- and *S*-metolachlor. The role of GSTs in the detoxification of certain herbicides has been known for many years. The activation of GST genes in response to biotic and abiotic stresses has also been reported previously [34], [35]. The non-selective up-regulation of *ZmGST27* expression by *rac*- and *S*-metolachlor is likely to be a general response of this gene to chemical stress.

Our earlier work has shown that *ZmGT1* is able to mediate the uptake of both GSH and GS conjugates by yeast cells, suggesting a potential role of this gene in xenobiotic detoxification [25]. The fact that *S*-metolachlor, which is enantiomerically enriched with the biologically active 1′*S*-isomer, gives rise to similar level of up-regulation of *ZmGT1* expression in comparison to *rac*-metolachlor implies that this glutathione transporter has no preference in transporting GS conjugates of *R*- and *S*-isomer of metolachlor.

It is known that ABC transporters of plants have a wider spectrum of substrates as compared to glutathione transporters [27] and they are able to transport GS conjugates as well as glucose conjugates of certain herbicides [36]–[38]. A smaller increase in *ZmMRP1* transcripts by *S*-metolachlor as compared to *rac*-metolachlor suggests that ABC transporters may preferentially transport GS conjugates of *R*-isomers. This may cause accelerated degradation of *R*-isomers, leading to reduced herbicidal activity of *rac*-metolachlor as compared to *S*-metolachlor. The dramatic decrease of EC_50_ value of *rac*-metolachlor and the quasi-equal growth inhibition effect between *rac*- and *S*-metolachlor in the presence of an ABC transporter inhibitor (vanadate) further support this hypothesis. However, further investigation is needed to obtain direct evidence for faster degradation of *R*-isomers. More work is also needed to find whether the stronger herbicidal activity of *S*-isomer is related solely to its slower metabolism in plants.

In summary, racemic metolachlor is currently being replaced by *S*-metolachlor in application around the world. Through comparing the expression of *ZmGST27*, *ZmMRP1* and *ZmGT1* after treatment by *rac*- and *S*-metolachlor, we have found that *rac*- and *S*-metolachlor display differential activation on one of the detoxifying genes, suggesting a possible link between ABC transporter activity and differential plant sensitivity to chiral herbicides.

## Materials and Methods

### Chemicals

*Rac*-metolachlor (95% purity) and *S*-metolachlor (96% purity) were kind gifts from Institute of Plant Protection, Chinese Academy of Agricultural Sciences. Sodium orthovanadate was purchased from Sinopharm Chemical Reagent Beijing Co., Ltd.

### Plant Material

Maize (*Zea mays*, cv Bainuo No.2) were grown on sand in the glasshouse as described previously [25]. Plants grown to the 3-leaf stage were transferred to a hydroponic culture (three plants per pot). Each pot contained 50 mL of nutrient solution. Plants were adapted for 7 d and the nutrient solution was changed every 2 d. After the 7 d adaptation period, *rac*- and *S*-metolachlor were added into the nutrient solution to a final concentration of 100 µM and the nutrient solution was changed daily. At the end of the treatment, leaves were harvested, immediately frozen in liquid nitrogen, and stored at −80°C.

### RNA Isolation

Frozen leaf samples were ground into a fine powder in liquid nitrogen. Total RNA was isolated using TRIzol reagent (Invitrogen, USA), and then digested by DNase I (TakaRa, China). First strand cDNA was synthesized with AMV reverse transcriptase and Oligo(dT)_15_, according to the manufacturer’s protocol of Reverse Transcription System (Promega, USA).

### Semi-quantitative RT-PCR Analysis of Transcripts

Three primer pairs (*ZmGST27-F* 5′- GAC CTG CTC CTC GCC TCC AA -3′and *ZmGST27-R* 5′- CCT CCA GCG TGT CCA TAG CG -3′; *ZmGT1-F* 5′- GTG CCG CAG TGG TGG TTC -3′ and *ZmGT1* 5′- GTG ACG ACG AAG GCG AGC -3′; *ZmMRP1-F* 5′- CTA GAA TAT GAA ACA CCA GCC AAG -3′and *ZmMRP1-R* 5′- CTG CAA TAA TGG TAG ATC ATG TTG -3′) were designed from the ORF region of *ZmGST27* (accession number AF244692), *ZmGT1* (accession number FJ573212), and *ZmMRP1* (accession number AY186244) respectively to amplify a single fragment for each gene. Another pair of primers (P3 5′- GCT CTT TCT TGA TTC TAT GGG TGG -3′ and P4 5′- GTT AGC AGG CTG AGG TCT CGT TC -3′) was used to amplify a fragment from maize 18S ribosomal RNA (accession number M82386), chosen as an internal reference. PCR amplification conditions were: 30 s at 95°C, 30 s at 62°C, 30 s at 72°C (30 cycles).

### Growth Inhibition Tests

The effect of *rac*- and *S*-metolachlor on the growth of maize shoots was assessed using the dose-response test. *Rac*- and *S*-metolachlor were dissolved in methanol to make a stock solution (10,000 mg L^−1^). The stock solution was then diluted to five serial concentrations (25, 50, 100, 200 and 400 µM) using distilled water. Triton X-100 was added at the rate of 0.5 mL L^−1^ as a wetting agent. Maize seeds were sown in plastic pots (9 cm diameter, 10 seeds per pot) filled with sand (250 g). After applying herbicide solutions, the pots (three replicates for each concentration) were placed in a growth chamber (25±2°C, 80±5% relative humidity) and watered as needed. Shoot lengths were measured 4 d after sowing. A 2^nd^ dose-response test of *rac*-metolachlor and *S*-metolachlor was performed in parallel in the presence of an ABC transporter inhibitor, vanadate (sodium orthovanadate), which was added to the herbicide dilutions to a final concentration of 100 µM. Controls were treated with vanadate only. The effective concentration of herbicide causing 50% reduction (EC_50_) in shoot length was determined from the dose-response curve by Probit Analysis using the SPSS (version 16.0).
